# Block Copolymer Elastomer with Graphite Filler: Effect of Processing Conditions and Silane Coupling Agent on the Composite Properties

**DOI:** 10.3390/polym10010046

**Published:** 2018-01-04

**Authors:** Denis Mihaela Panaitescu, Raluca Augusta Gabor, Cristian Andi Nicolae, Anca Constantina Parau, Catalin Vitelaru, Valentin Raditoiu, Mircea Chipara

**Affiliations:** 1 Polymer Department, National Institute for Research and Development in Chemistry and Petrochemistry, 202 Spl. Independentei, Bucharest 060021, Romania; raluca.gabor@icechim-pd.ro (R.A.G.); cristian.nicolae@icechim-pd.ro (C.A.N.); vraditoiu@icechim.ro (V.R.); 2 National Institute for Optoelectronics INOE 2000, 409 Atomistilor St., Magurele 077125, Romania; anca.parau@inoe.ro (A.C.P.); catalin.vitelaru@inoe.ro (C.V.); 3 Department of Physics and Astronomy, The University of Texas Rio Grande Valley, 1201 W. University Drive, Edinburg, TX 78539, USA; mircea.chipara@utrgv.edu

**Keywords:** block copolymer, surface treatment, graphite, vinylsilane

## Abstract

The control of morphology and interface in poly(styrene-ethylene/butylene-styrene) (SEBS) composites with graphitic fillers is extremely important for the design of piezoresistive sensors for body motion or flexible temperature sensors. The effects of a high amount of graphite (G) and silane coupling agent on the morphology and properties of SEBS composites with anisotropic mechanical properties are reported. The physical and chemical bonding of silane to both G and SEBS surface was proved by EDX and TGA results; this improved interface influenced both the thermal and mechanical properties of the composite. The vinyltriethoxysilane (VS) promoted the formation of char residue and, being tightly bound to both SEBS and G, did not show separate decomposition peak in the TGA curve of composites. The mechanical properties were measured on two perpendicular directions and were improved by both the addition of VS and the increased amount of G; however, the increase of storage modulus due to orientation (from 5 to 15 times depending on the composition and direction of the test) was more important than that provided by the increase of G concentration, which was a maximum of four times that obtained for 15 wt % graphite. A mechanism to explain the influence of G content and treatment on the variation of storage modulus and tan δ depending on the direction of the test was also proposed.

## 1. Introduction

Carbon-based fillers are commonly used in polymeric matrices to improve their mechanical, tribological, thermal, and electrical properties or to produce new functionalities [1,2]. Graphite (G) is a low-cost filler that can be used in elastomers to increase the electrical conductivity and mechanical properties. Graphite and graphitic fillers (expanded graphite, graphite nanoplatelets, and graphene) are among the most studied fillers for elastomers due to their striking properties [1,2,3,4,5]. However, the extent of property improvement depends on the size and aspect ratio of the filler, the degree of dispersion and orientation in the matrix, and the polymer-filler interface strength [1,2,3,4,6]; smaller size, higher aspect ratio, and good interface promote better dispersion and properties of the elastomeric matrix.

Several physical and chemical methods were proposed for the conversion of graphite to nanofillers, with graphite intercalation approaches being the most used [1]. The mechanical exfoliation was less studied, and only mechanical milling and sonication were reported to break the van der Waals binding and to release graphene sheets [1,4,7,8]. Nevertheless, recent work has shown that graphite flakes are disintegrated in submicron particles due to the shear forces during the melt processing of block copolymer elastomers with graphite [9]; AFM investigation of SEBS and maleic-anhydride-grafted SEBS (SEBS-MA) composites with 5 wt % graphite showed that graphene sheets can be obtained in-situ during the melt processing, leading to a simultaneous increase in tensile strain, strength, and Young’s modulus compared to pure matrices [9]. The in-situ release of graphene layers during the processing step is important, because in the common melt mixing of polymer matrices with graphene a decline of the proprieties was reported due to the self-reaggregation tendency of graphene [1,5]. Moreover, graphite is an abundant, low-cost filler, and the melt compounding technique is a high productivity, eco-friendly, well-established procedure.

Block copolymer elastomers are a special group of elastomers, consisting of physically cross-linked glassy and rubbery microdomains. The morphological characteristics (microdomains, orientation) have a great influence on the physical and mechanical properties of block copolymers, and various methods were employed to control and improve these properties [3,10,11,12,13,14]; for example, multigraft copolymers with tetrafunctional branch architecture reached a huge strain at break depending on the molecular architecture [12,13]; likewise, orientation induced during the manufacturing process determined anisotropic mechanical properties in SEBS block copolymers and their composites with G [11]. Recently, a composite film containing polyurethane elastomer and surface treated graphite, prepared by solution casting, was proposed as a promising material for piezoresistive sensors for body motion, wearable devices for human healthcare, and garment pressure testing [15]; a γ-aminopropyl-triethoxy silane was used for the surface treatment of graphite. Similarly, the improvement of graphite–SEBS elastomer interface is of utmost importance for such applications for a better control of electrical and mechanical properties. Only a few attempts have been made to improve the SEBS–G interface [9,16]. In one attempt, both π–π interaction and hydrogen bonding were created in a SEBS–graphene oxide composite by sulfonating SEBS block copolymer (SSEBS). The strong π–π interaction between the benzene groups in SSEBS and graphene oxide nanosheets and hydrogen bonding between hydroxyl or carboxyl groups of graphene oxide and sulfonic groups of SSEBS highly improved the stress transfer and the mechanical performance of the composites [16]. In our previous work [9], maleic anhydride grafted SEBS was used instead of SEBS in composites with graphite, showing better mechanical behavior due to the improved interfacial adhesion by π–π interactions between styrene blocks and graphitic planes, and hydrogen bonds between maleic anhydride and the oxidized groups from the surface of the graphene sheets.

Silane coupling agents are well known for their ability to improve the polymer-filler interface [15,17,18]. Most of the studies concerning silane-treated carbon and graphite fillers are in the case of composites with thermosetting matrices [18]. No study on the influence of silane coupling agents in SEBS composites with graphitic fillers was reported, although the improvement of the polymer–graphite interface is very important for highly stretchable, sensitive, and flexible sensors [15]. SEBS block copolymer undergoes microphase separation into self-assembled ordered morphologies as a result of the incompatibility between the blocks constituting the copolymer, and the control of these morphologies in nanometers range is important for these applications. A small amount (5 wt %) of graphite diminished the anisotropy of the mechanical properties in SEBS composites obtained by the melt processing technique [11]. To manipulate the design of these elastomeric composites, the effect of a higher amount of G and silane coupling agent on the morphology and properties of SEBS composites with anisotropic mechanical properties is reported. G concentration was chosen between 5 and 15 wt %; a higher concentration of G has not been considered, because some of the mechanical properties including elongation and stretching ability are rapidly decaying above the percolation threshold (*p*), when a contiguous path of the conductive filler is formed in the composite [19]. *p* values of 15–20 wt % were reported for polyolefin composites with micrometric or sub-micrometric size graphite [20,21] and around 7 vol % (about 15 wt %) for PP/graphite nanoplatelets composites [22].

## 2. Materials and Methods

### 2.1. Materials

SEBS block copolymer (Kraton G1652MU, 29.9 wt % styrene, with density of 0.910 g/cm^3^ and Hardness-Shore A of 69) was supplied by Kraton Polymers (Houston, TX, USA). G powder, with particles smaller than 75 µm, was supplied by Georg H. Luh. Gmbh (Walluf, Germany). Sterically hindered phenolic antioxidant (Irganox 1010) from Ciba-Geigy (Basel, Switzerland) was used as stabilizer, and vinyltriethoxysilane (VS) purchased from Dow Corning (Midland County, MI, USA) was used as coupling agent.

### 2.2. Preparation of Composites

SEBS block copolymer was melt-mixed with a different amount of graphite (from 0 wt % to 15 wt %) in a Brabender mixer (Duisburg, Germany) at 60 min^−1^, in the following temperature regime: 175 °C for 3 min (SEBS melting), rapid decrease at 160 °C (addition of G and VS), and mixing at this temperature for another 7 min. The treatment with VS was carried out “in situ” in the batch mixer at the same time with the dispersion of G in the polymer matrix. This method is commonly used in practice [23]. The composites were then molded on a laboratory two-roll-mill heated to 110 °C for 90 s and compression molded into sheets of 0.5 mm in thickness at 175 °C, 3 min preheating (0.5 MPa), and 2 min under pressure (15 MPa). The composites with G were denoted as SEBS-xG, where x is the weight concentration of G, and that with G and VS were denoted as SEBS-xGVS with the same notation for x.

### 2.3. Characterization

Thermogravimetric analysis (TGA) was performed on a TA-Q5000 V3.13 (TA Instruments, New Castle, DE, USA) between 25 °C and 700 °C at a heating rate of 10 °C/min. Nitrogen was used as the purge gas at a flow rate of 40 mL/min. The error was ± 0.5 °C for the temperature.

Dynamic mechanical analysis (DMA) experiments were performed in tensile loading mode using a DMA Q800 (TA Instruments) at a constant frequency of 1 Hz with oscillation amplitude of 4 μm. The storage modulus, loss modulus, and loss factor (tan δ) were recorded as a function of temperature from room temperature (RT) to 150 °C with a heating rate of 3 °C/min and force track of 125%. The samples, 16 × 6.2 × 0.7 mm (length × width × thickness), were cut from compression molded plates, parallel (II) and perpendicular (⊥) to the rolling direction. All samples were equilibrated at RT for 5 min. Duplicate samples were tested for each measurement.

The morphology and the elemental composition on the surface of composites was analyzed by scanning electron microscopy (SEM) using a Tabletop Microscope TM 3030PLUS (Hitachi, Tokyo, Japan) equipped with energy dispersive X-ray spectrometry (EDX) system Quantax70 (Bruker, Billerica, MA, USA) at an accelerating voltage of 15 kV. The identification and quantification of atomic elements was done on 3 parallel samples for each composite and for the reference. The surface area under investigation was about 1700 μm^2^ and no coating was applied. SEM images of fractured samples (in liquid nitrogen) were also collected. Density was determined with a pycnometer.

Fourier transform infrared (FTIR) spectra were obtained with a Tensor 37 spectrophotometer from Bruker (Billerica, MA, USA), with attenuated total reflectance accessory. FTIR spectra were recorded in absorbance mode, at room temperature, in the mid infrared range (4000 to 400 cm^−1^), with 16 scans at a resolution of 4 cm^−1^. FTIR data were collected on duplicate samples. The spectra were normalized using the peak at 2920 cm^−1^ (C–H vibration) for easier comparison.

## 3. Results and Discussion

### 3.1. The Effect of Silane on SEBS-G Composites

**Figure 1:**
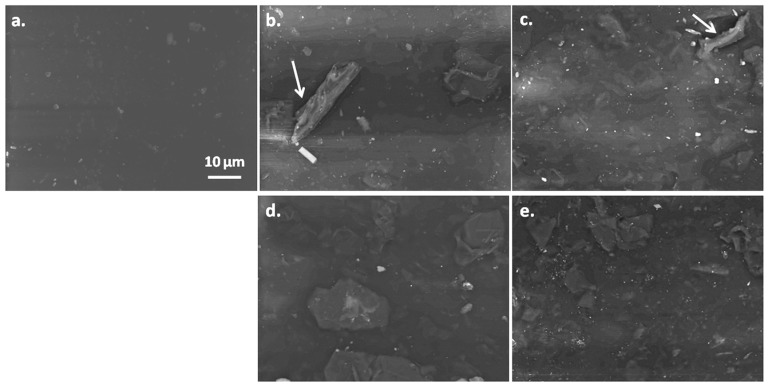
SEM images of SEBS (**a**) and composites: SEBS-10G (**b**); SEBS-10GVS (**c**); SEBS-15G (**d**); SEBS-15GVS (**e**).

**Figure 2:**
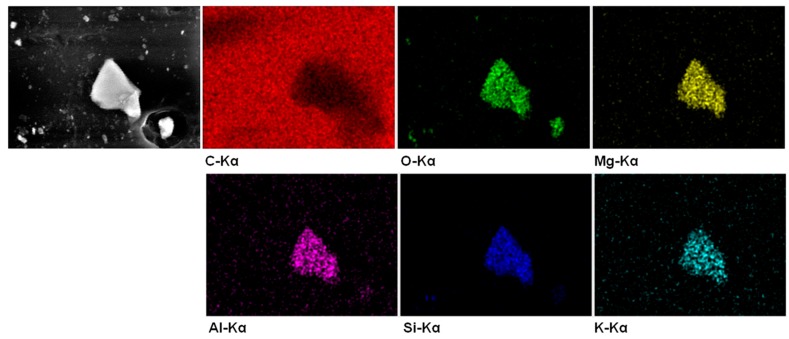
EDX analysis of the filler on the surface of SEBS.

SEM micrographs on the surface of SEBS and SEBS-G composites are illustrated in Figure 1. Some light-colored, micron-size objects were observed on the surface of SEBS matrix (Figure 1a). These may come from the two-phase morphology of SEBS, containing polyethylene–butylene (EB) and polystyrene (S) blocks, or from the fillers used in the commercial SEBS matrix. EDX elemental maps of such an object on the surface of SEBS (Figure 2) clearly show that this is a filler, namely an aluminosilicate also containing Mg and K elements. Less bright particles of different sizes, ranging from less than one micron to more than 10 µm, were observed only on the surface of composites and they were assigned to G particles embedded in the block copolymer (Figure 1b–e). The dispersion of G was estimated using around five SEM images for each composite. A better dispersion was observed for the silane treated samples.

The EDX analysis on whole images of composites provided the surface composition (Table 1). Only the weight percentage of C and O were shown for SEBS matrix, because the amount of aluminosilicate elements was small and the concentration of the Mg, Al, Si, or K (Figure 2) fell within the range of experimental error.

**Figure 3:**
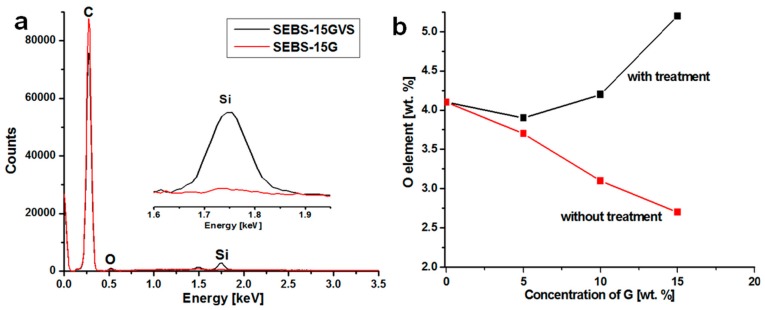
EDX spectra of SEBS-15GVS and SEBS-15G showing almost no Si on the surface of the untreated composite (**a**); wt % of O element on the surface of composites (**b**).

The presence of Si was detected only on the surface of the samples treated with silane and containing 10 and 15 wt % G (Table 1, Figure 3a), because the amount of the added silane was proportional to the amount of added G. The concentration of Si fell within the range of experimental error for the composite with silane treatment and 5 wt % G because of the low concentration of the added silane.

The presence of O in the SEBS matrix may come from the aluminosilicate filler or from oxidative processes. Smaller amount of O element was detected on the surface of untreated SEBS-G composites compared to that of SEBS (Figure 3b) due to the addition of G and the percentage of oxygen decreased with the increase of G content in SEBS, as expected (Table 1). On the other hand, higher proportion of O was obtained for silane-treated composites compared to the untreated ones due to the oxygen content of silane (Figure 3b). The concentration of added VS was proportional to that of G, but the increase of the percentage of O determined by EDX was not proportional to the increasing amount of VS (Table 1); this may suggest different interactions between VS and SEBS or G.

**Figure 4:**
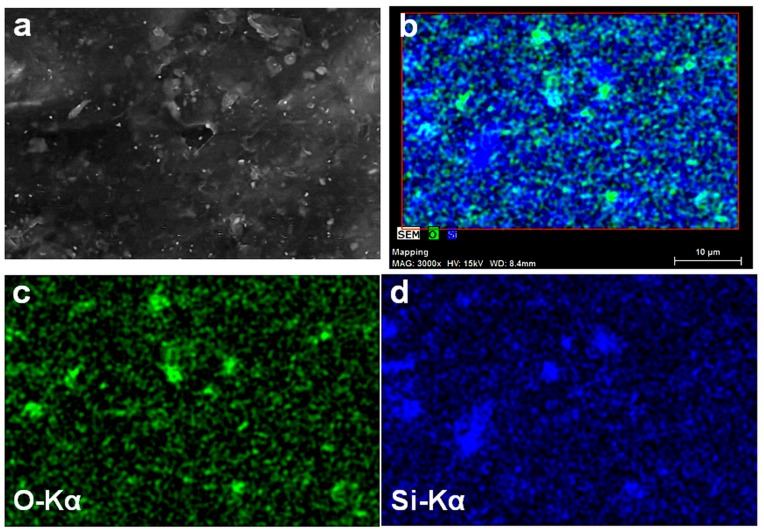
SEM image of SEBS-15GVS (**a**); Combined map of O and Si elements (**b**); O Kα and Si Kα maps of Figure 4a (**c**,**d**).

The location of O and Si elements on the surface of SEBS-15GVS may provide more information (Figure 4). Indeed, SEM image (Figure 4a) shows both aluminosilicate and G particles on the surface of the composite, which differ in size and brightness, but O Kα and Si Kα maps show both common and different locations of these elements (Figure 4b–d). Considering that the atomic proportion of O in silane is lower than that in aluminosilicate, the locations rich in O will indicate the aluminosilicate particles and that rich in Si, the silane bond to the filler/matrix or as oligomeric polisiloxane. Interestingly, most of the locations rich in Si overlap with G particles, which generally appear less bright in the SEM image (Figure 4a). This means that, although the silane is well dispersed in the composite (Figure 4d), it has higher affinity for G particles.

The commonly accepted mechanism of silane action states that the ethoxysilane (Si–OEt) moiety of VS is hydrolyzed in the presence of moisture, forming more reactive silanol group that binds to the surface of the filler or gives rise to Si–O–Si bonds in cyclic or linear oligomers because of self-condensation [24]. However, some features differentiate SEBS-GVS composites, obtained by the addition of VS in the melt-mixing step. Firstly, the amount of water in both SEBS and G is very small so that the hydrolysis of Si–OEt moieties of VS is not very likely; TGA results showed that the weight loss of SEBS at 160 °C (the processing temperature) was almost 0 and that of G was close to 0.13%; at this temperature, the free and bound water contained in the samples should be released. Secondly, G surface is not rich enough in OH, C=O, or COOH to react with the Si–OH or Si–OEt groups of VS, although their presence on the surface of graphene was reported [25]; however, the aluminosilicate filler in reasonable amount may provide Si–OH and Al–OH groups to react with the silanol groups of VS, but the TGA analysis of SEBS matrix showed a residue at 500 °C of only 0.16%, and therefore a very small amount of inorganic filler. If the oxygen detected by EDX in SEBS (Table 1) is coming from oxidative processes, then COOH, OH, or epoxy groups are present on the surface of SEBS, and hydrogen bond interactions between these groups and Si–OH or Si–OEt from VS may be expected.

**Figure 5:**
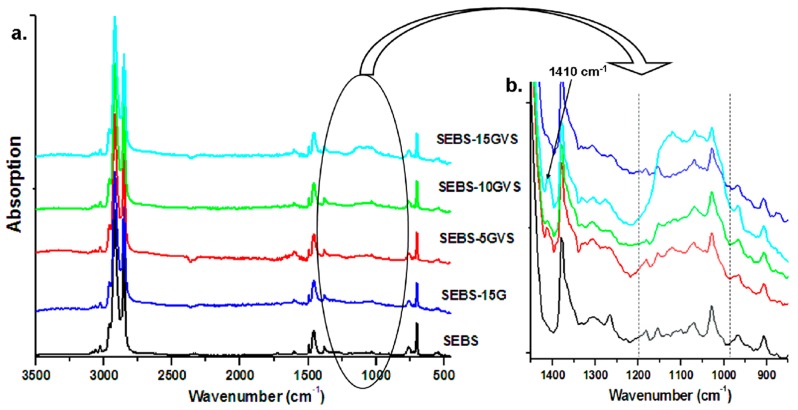
FTIR spectra of SEBS-G composites treated with silane (**a**); SEBS and SEBS-15G spectra are given for comparison; FTIR spectra in the range from 1450 cm^−1^ to 850 cm^−1^ (**b**).

FTIR analysis may give more information on these aspects. The overlapped FTIR spectra of composites are shown in Figure 5a. The highest difference was detected in the range from 1000 cm^−1^ to 1200 cm^−1^, and this was detailed in Figure 5b.

Higher intensity of the vibrations in the range from 1000 cm^−1^ to 1200 cm^−1^, characteristic of Si–O–Si/Si–O–C bonds [26,27,28,29], was observed with the increase of G concentration (and VS amount) in composites (Figure 5b). A thorough analysis of this region provides more evidence of the VS chemical bonding. The strongest new peak and shoulder in the FTIR spectra of SEBS composites containing VS are in the range from 1050 cm^−1^ to 1150 cm^−1^, and are characteristic of VS bound to the polymer matrix, probably by the EB blocks (Si–O–CH_2_–) [22,25]. The presence of a shoulder at 1053 cm^−1^ in silane treated samples, characteristic of Si–O–Si asymmetric stretching vibration [28,29], confirms the condensation reaction of Si–OH and/or Si–OEt groups with the formation of oligomers or silsesquioxane cages [30]; thus, radical reactions may be thermally activated due to the high processing temperature, and VS can form cyclic or linear oligomers [30]. The Si–OH group absorbs at 800–950 cm^−1^, but no new peaks or intensity increase were observed in this region after silane treatment; this shows that either the hydrolysis of VS was not very extensive due to the special reaction conditions, or all silanol groups were condensed in Si–O–Si linkages. These observations are consistent with TGA results, which showed only small amount of released water in both SEBS and G.

The role of the vinyl group in VS seems to be very active in the melt processing conditions. Indeed, the small peak at 1603 cm^−1^ was broadened to lower wavenumber in silane treated samples; this peak is characteristic to –C=C stretching [30], and it also occurs in SEBS and SEBS-G without VS due to styrene blocks, but it is modified as intensity and wavenumber in treated composites; this could mean new π–π interactions between the vinyl group in VS and the aromatic side chains of SEBS, on the one hand, and the condensed rings in the graphene layers of G, on the other hand. The processing temperature during the preparation of the samples was high (close to 160 °C), and the occurrence of free radicals cannot be avoided due to mechano-chemical processes; these free radicals may favor the grafting of VS to both phases [31]. Interestingly, a small peak was observed at about 1410 cm^−1^ in silane treated samples and corresponds to the vinyl group in VS [26]; the intensity of this peak was small in SEBS-5GVS and SEBS-10GVS but slightly higher in SEBS-15GVS (Figure 5b), the composite with the highest concentration of silane. This shows that when used in high amount, VS was not entirely bond to the surface of SEBS or G by its vinyl moiety.

In conclusion, FTIR data showed the presence of cyclic or linear oligomers with Si–O–Si linkages and the occurrence of π–π interactions between the vinyl group in VS and the condensed rings in G or the aromatic rings in SEBS. If the silane is anchored to both G and SEBS surface by its vinyl groups, the oligomerization of the silane is beneficial to SEBS-G composites, because it allows VS to act as a bridge between the polymer matrix and G.

### 3.2. The Effect of G and VS on the Thermal Behavior of Composites

**Figure 6:**
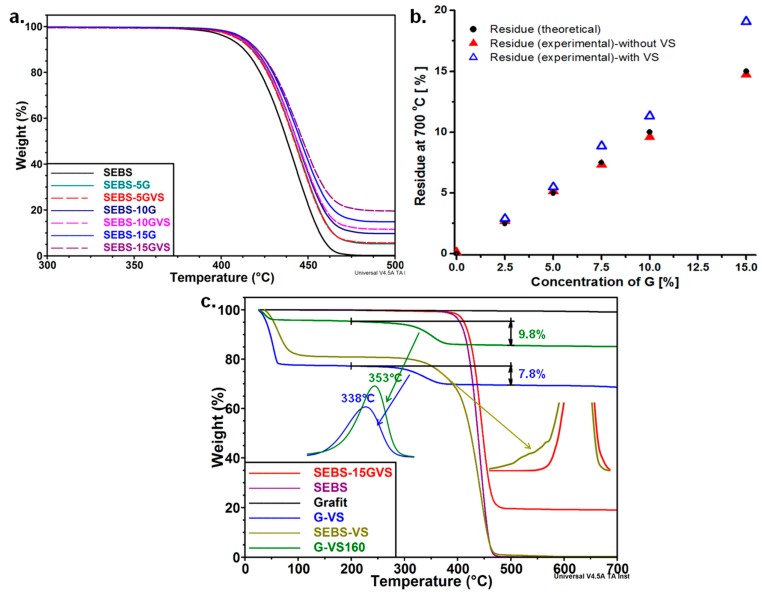
TGA diagrams of SEBS-G composites with and without silane treatment (**a**); theoretical and experimental values for the residue at 700 °C (**b**); TGA curves for G-VS and SEBS-VS (**c**).

The TGA curves of SEBS-G composites are shown in Figure 6a. The addition of G has small influence on the thermal degradation of SEBS under nitrogen atmosphere. This was expected, because SEBS is characterized by a high thermal stability, with less than 0.5% weight loss (WL) up to 300 °C. The values of *T*_20%_ (the temperature corresponding to 20% WL) slightly increased with the amount of G, and the highest increase was of 7.3 °C for the *T*_20%_ of SEBS-15G compared to that of SEBS (Table 2). G addition slightly enhanced the thermal stability due to the physical barrier effect of graphene layers [32] that hinder the propagation of decomposition reactions and promote the char formation [26].

It is worth mentioning that less than 0.5% WL was noticed up to 300 °C for all the samples, but at a higher temperature the differences were obvious; more than 77% of the initial mass was lost up to 450 °C in the case of the SEBS matrix and only 58.6% for SEBS-15G. Interestingly, the highest improvement relative to the amount of G was noticed for SEBS-5G; only 5 wt % G reduced the WL at 450 °C from 77.6% to 67.1% and increased *T*_20%_ from 422.4 °C (SEBS) to 426.7 °C (SEBS-5G).

The addition of VS had no obvious influence on the degradation temperature of SEBS-G composites; *T*_20%_ was similar for the samples with the same amount of G regardless of the treatment. However, an important increase of the char residue was noticed with the increase of the concentration of G and VS (Figure 6b).

The silane may influence the thermal stability of the composites by inducing interactions between SEBS and G particles and by promoting the formation of char residue; both the physical and chemical bonds of VS to the filler or matrix will reduce the segmental mobility and decrease the diffusion rate of volatile by-products [33]. More insight into the influence of VS on the degradation of SEBS-G composites may be gained by comparing the theoretical and experimental values of the residue. The concentration of G in SEBS-G composites was considered the theoretical value of the residue (Figure 6b), because the weight loss of SEBS at 700 °C is close to 0 (Table 2), and G remains almost unaffected by temperatures up to 700 °C (Figure 6c). The experimental values of the residue for the composites without silane were close to the theoretical values, but, after the addition of VS, the residue was higher, especially for the samples with more G and, thus, contained more silane (Figure 6b); therefore, the increased residue is an effect of silane and shows the ability of VS to enhance the interaction between G and SEBS, and the amount of char.

Usually, the decomposition of VS occurs between 150 °C and 370 °C [27], but the influence of the environment could modify this range. However, the decomposition of SEBS-GVS composites was one-step process, and no degradation step corresponding to VS was observed in TGA diagrams in this range or outside of it. This suggests that most of the silane is tightly bound to SEBS, or SEBS and G, so that its degradation occurs in the same range with that of the polymer.

To verify this hypothesis, several mixtures were made to simulate the conditions used for the preparation of the composites: SEBS-VS 1:1, G-VS 1:1, and G-VS160 (G-VS 1:1 treated at the same temperature and for the same period as in the experimental part). The G:VS ratio was chosen higher than in the real tests to better catch the influence of silane. G-VS sample begin to lose weight from less than 100 °C (Figure 6c), probably due to the evaporation of VS and its partial hydrolysis; yet, only a part of the silane was lost at this stage (about 20%). At a higher temperature, a second step was noticed; about 7.8% of weight was lost between 250 °C and 400 °C, with a maximum degradation rate (*T_m_*) at 338 °C. This is probably determined by the reactions of VS, self-condensation, cyclization, and bonding to G surface, as shown by FTIR results. Interestingly, 9.8% of weight was lost in the same range of temperature in the case of G-VS160 with a maximum degradation rate at *T_m_* = 353 °C; the increased weight loss and higher *T_m_* suggest a higher proportion of VS bond to G surface after the treatment at 160 °C. This highlights once more that VS was bound to the surface of G during the melt processing of composites. The residue in G-VS samples probably consists of polysiloxanes, which are cross-linked at this temperature. On the other hand, SEBS-VS mixture decomposed faster than SEBS; the decomposition of VS started before that of SEBS and appeared as a shoulder in the DTG diagram (Figure 6c) but only slight increase of the residue was observed; this shows that the degradation products of VS were released together with that of SEBS, and, therefore, VS was bound to the SEBS matrix. No shoulder was detected in the DTG diagram of SEBS-15GVS. The lack of the shoulder and the higher thermal stability of SEBS-15GVS compared to that of SEBS-G mixture or pure SEBS highlight the influence of G, which favors the physical and chemical bonding of VS to both filler and matrix. This is in good agreement with FTIR results, which showed that VS is bonded to G and SEBS by its vinyl functions, thus enhancing the thermal properties of the composite.

### 3.3. Mechanical Behavior by DMA Analysis

**Figure 7:**
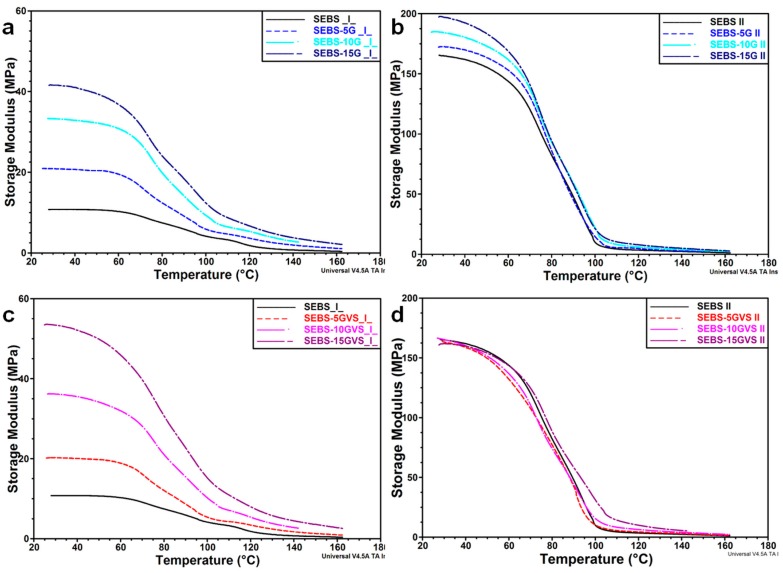
Storage modulus of composites and VS treated composites vs. temperature in a perpendicular (**a**,**c**) and parallel (**b**,**d**) direction to the orientation induced during the manufacturing process.

**Figure 8:**
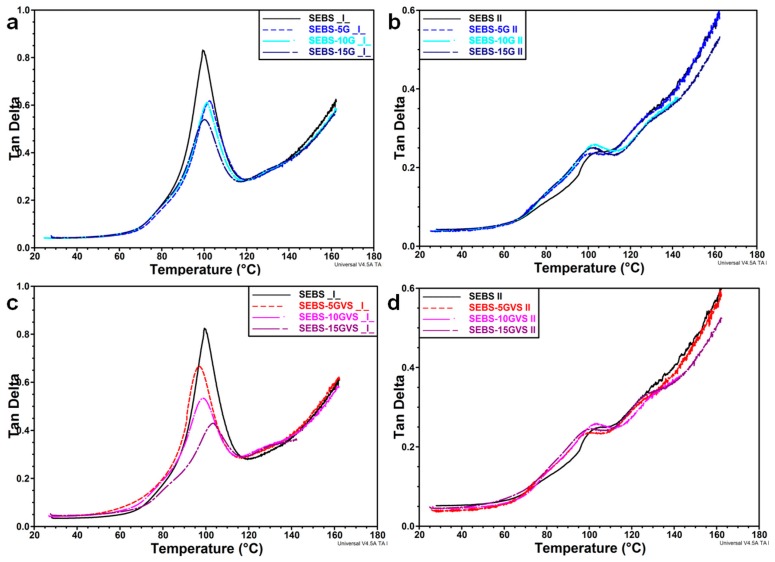
Tan δ of composites and VS treated composites vs. temperature in a perpendicular (**a**,**c**) and parallel (**b**,**d**) direction to the orientation induced during the manufacturing process.

Depending on the processing conditions, SEBS shows anisotropic mechanical properties in the stretching direction and in the perpendicular one [11]. Diminished anisotropy was induced by the addition of only 5 wt % G in SEBS. Therefore, the addition of a coupling agent and of higher amount of G may also influence the mechanical behavior of these composites. To investigate the mechanical behavior of SEBS-G and SEBS-GVS composites, dynamic mechanical analysis was carried out from room temperature to 160 °C, and storage modulus (*E*’) and tan δ are shown in Figure 7 and Figure 8. Depending on the test direction and G content, the values of *E*’ at room temperature were very different: from 11.0 ± 0.2 MPa for SEBS to 40.7 ± 0.9 MPa for SEBS-15G for the perpendicular test and from 166.3 ± 1.3 MPa to 197.8 ± 0.6 MPa for the same samples in the parallel test (Figure 7a,b). The highest increase of *E*’ value due to G addition was four times and was obtained for the perpendicular test, and the increase of *E*’ due to orientation varied from 5 to 15 times depending on the composition and direction of the test. Therefore, the improvement of *E*’ due to orientation was more important than that provided by the increase of G concentration.

With the increase of the temperature, the modulus decreases sharply and begins to stabilize at about 100 °C for the parallel test and at a higher temperature in the case of the perpendicular test. These differences occur due to the oriented polystyrene domains as also noticed in [11]. A lamellar or cylindrical morphology is, generally, assumed for the S blocks in SEBS [11,34]. During the manufacturing process, the lamellar/cylindrical S blocks are oriented in the rolling direction also engaging the EB mid-blocks to which they are covalently linked. During the mechanical test in a direction perpendicular to that of the oriented blocks in SEBS (Figure 7a), the flexible EB blocks are stretched, increasing the separation between S blocks. In the composites, the stiff G particles, mostly dispersed in the rubbery phase, will restrict the flexibility of polymer chains, increasing the storage modulus [34,35]. The π–π interactions between S blocks and graphitic planes [9] will also decrease the mobility, increasing the constraints. These effects will be enhanced by a higher concentration of G (Figure 7a).

The use of a coupling agent (Figure 7c) will improve the dispersion of G particles and will further reduce the mobility of molecular chains by tightly bound them to the surface of G. However, for low amount of VS, the differences were not significant, and the increase of *E*’ value was observed mainly for higher amount of coupling agent; thus the *E*’ value of SEBS-15GVS was with about 30% higher than that of SEBS-15G (Table 3).

For the test in a direction parallel to that of oriented blocks in SEBS, the S and EB blocks are already stretched in the direction of the test, and higher energy is necessary to stretch the elongated EB blocks and the rigid and oriented S blocks; this has, as result, a very large *E*’ value, 15 times higher than that in the perpendicular direction. The addition of G restricts the mobility of the molecular chains, thus increasing the storage modulus, but the increase of *E*’ value in II compared to ⊥ direction is smaller in composites compared to pure SEBS (Table 3); this shows that higher concentration of G reduces the effect of orientation on the increase of storage modulus (Figure 7b). The use of the coupling agent (Figure 7d) will result in additional bonding between G particles and SEBS blocks, which will hinder the orientation of S blocks during the manufacturing process; therefore, the effect of a higher concentration of stiffer graphitic filler will be counterbalanced by a lower orientation, resulting in no variation of *E*’ value for SEBS-GVS composites in the II direction. Moreover, it may be assumed that the oligomeric silanes, emphasized by FTIR, will act as lubricants and will decrease the stiffness.

A main peak at about 100 °C was observed in the tan δ curve for the perpendicular direction regardless of G concentration and treatment (Figure 8a,c). This is assigned to the glass transition of S blocks. The intensity of this peak was lower in the composites compared to SEBS matrix, showing an increased rigidity (Figure 8a,c). The peak value of tan δ was only slightly influenced by G concentration in untreated composites, but gradually decreased in SEBS-GVS composites, which is probably an influence of the silane treatment. Some small peaks were observed before and after this main peak only in composites and were more obvious after VS treatment. They can be related to the S blocks differently bonded to G. For example, better interactions are expected in VS treated samples, and G particles may differently interact with S blocks depending on their nano- or microsize [3]. For the test in the parallel direction, the variation of tan δ values with the amount of G falls within experimental error for all the composites. It is worth mentioning that the intensity of the main peak is very small for both untreated and treated composites in the parallel test, and this shows constraints and high rigidity. This is caused by the induced orientation of S and EB blocks in the same direction as that of the test, determining high values of the storage modulus and, for similar loss modulus values, very low tan δ values.

### 3.4. Density and Morphology

**Figure 9:**
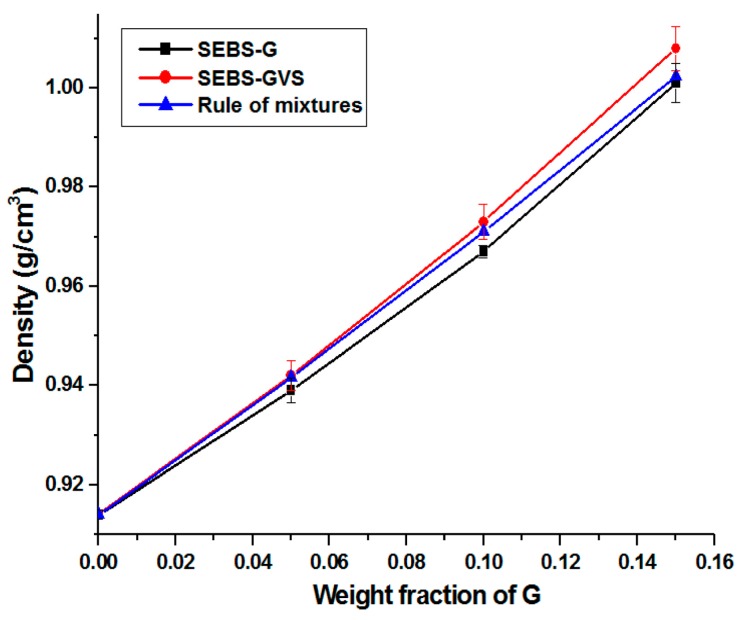
Density of SEBS-G and SEBS-GVS composites containing different amount of treated or untreated G.

In several studies, density was found as a strong determinant for the mechanical properties of polymer composites [36,37]. The measured values of density for SEBS-G and SEBS-GVS series are shown in Figure 9. Density of composites was calculated using the rule of mixtures [36], considering the density of G, 2.23 g/cm^3^ [38] and the density of SEBS, 0.914 g/cm^3^ (experimentally determined).

The density of the composites increased with the increase in G due to the higher density of G (double than that of the matrix), and the corresponding values for the treated and untreated composites are close to each other and close to that calculated with the rule of mixtures. However, the experimental values of density are higher than the calculated ones for SEBS-GVS composites with 10 and 15 wt % G. This can be explained by the good interactions between SEBS and G particles due to the silane treatment. Indeed, FTIR data pointed out π–π interactions between the vinyl group in VS and the condensed rings in G or the aromatic rings in SEBS, with the silane acting as a bridge between the polymer matrix and G. Good interactions also mean no voids at the interface, resulting in higher densities than those predicted by the rule of mixtures.

Slightly lower densities were obtained for the untreated samples, due to the poorer interface. It can be supposed that the insufficient covering of G with the polymer and the voids accumulated at interface are the causes of this decay [35]. Voids fraction (*V*, %) was calculated with the following equation:

$$V,\;\%=\frac{\rho_{c}-\rho_{exp}}{\rho_{c}}\times 100$$

where *ρ_c_* and *ρ_exp_* are the calculated and experimental values of density.

*V* values of 0.27, 0.42, and 0.14% were obtained for SEBS composites with 5, 10, and 15 wt % G in contrast to the treated composites, stressing the effectiveness of the silane treatment.

**Figure 10:**
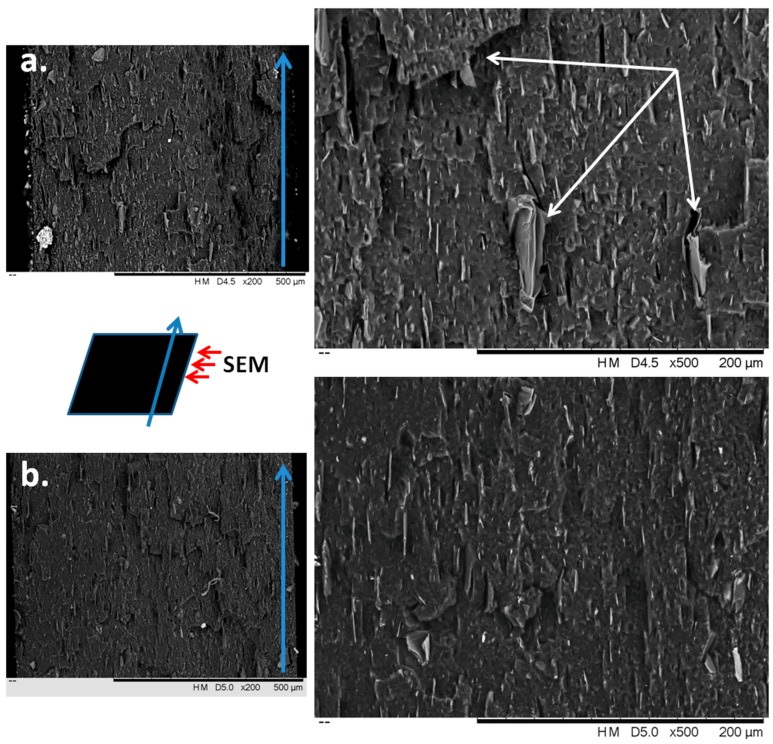
SEM images of fractured samples: SEBS-15G (**a**) and SEBS-15GVS (**b**).

The dispersion of G in SEBS-15G and SEBS-15GVS composites has been characterized by SEM at a low magnification on fractured samples (Figure 10).

The fractured plates were visualized on the whole width (Figure 10—left images) and at higher magnification (right images). It may be observed that G particles (lighter color) are in the form of platelets of graphene layers having a thickness ranging from tens of nanometers to a couple of microns. Therefore, some exfoliation of G occurred during the melt processing step. It is remarkable that most of the graphene layers are oriented regardless of their size and treatment, thus keeping the orientation induced during the manufacture. Some voids are clearly observed in SEBS-15G composite, especially surrounding larger G particles (Figure 10a—white arrow). Graphene platelets seem well dispersed in SEBS in both composites, but smaller size platelets (shorter and thinner) were observed in SEBS-15GVS compared to the untreated composite. These observations correlate well with DMA results, which showed a 30% increase of the storage modulus in SEBS-15GVS compared to SEBS-15G.

## 4. Conclusions

SEBS block copolymer undergoes microphase separation into self-assembled ordered morphologies. The processing-induced orientation affects the mechanical properties of nanocomposites. The block copolymer–graphite interface is also very important for highly stretchable parts and, therefore, the effect of 5–15 wt % G and the silane coupling agent on the morphology and properties of SEBS composites with anisotropic mechanical properties was reported. SEM, EDX, FTIR, and TGA were used to elucidate the influence of the vinylsilane treatment. Both cyclic or linear oligomers with Si–O–Si linkages and the occurrence of π–π interactions between the vinyl group in VS and the condensed rings in G or the aromatic rings in SEBS were observed; the physical or chemical linkage of silane to both the G and SEBS surfaces contributed to increased interaction between the polymer matrix and G. Moreover, the silane promoted the formation of char residue and, being tightly bound to both SEBS and G, did not show a separate decomposition peak in the TGA curve. The thermo-degradation of SEBS during melt processing was demonstrated through EDX by the presence of elemental O on its surface; the atomic proportion of O decreased proportionally to the increase of G content in untreated composites but increased in composites with a high amount of G and VS, showing the influence of both G and VS concentration. The addition of VS and the increased amount of G also influenced the mechanical behavior of composites. However, *E*’ improvement due to orientation was more important than that provided by the increase of G concentration. The highest increase of *E*’ value due to G addition was four times, being obtained for the perpendicular test, whereas the increase of *E*’ due to orientation varied from 5 to 15 times depending on the composition and direction of the test. A mechanism to explain the influence of G content and treatment on the variation of the storage modulus and tan δ depending on the direction of the test was also proposed. These results are important for the design of bidirectional sensors for artificial skin as an alternative solution to that of stacking two sensors oriented in different directions.

## Figures and Tables

**Table 1:** Elemental composition on the surface of SEBS and composites.

Sample	Elemental Composition (wt %) *		
	C	O	Si
SEBS	95.9	4.1	-
SEBS-5G	96.3	3.7	-
SEBS-5GVS	96.1	3.9	-
SEBS-10G	96.9	3.1	-
SEBS-10GVS	95.2	4.2	0.6
SEBS-15G	97.3	2.7	-
SEBS-15GVS	93.6	5.2	1.2

* The standard deviation was 9–10 and 0.4–0.5 for the wt % of C and O, respectively.

**Table 2:** TGA results.

Sample	*T*_20%_, °C	WL_450 °C_, %	Residue at 700 °C, %
SEBS	422.4	77.6	0.11
SEBS-5G	426.7	67.1	5.15
SEBS-5GVS	426.4	67.7	5.49
SEBS-10G	428.1	64.0	9.62
SEBS-10GVS	427.7	63.0	11.32
SEBS-15G	429.7	58.6	14.75
SEBS-15GVS	430.3	55.6	19.07

**Table 3:** DMA results.

Sample	*E*’ (⊥) 30 °C, MPa	*E*’ (II) 30 °C, MPa	*E*’ (⊥) 100 °C, MPa	*E*’ (II) 100 °C, MPa	*T*_g_ (⊥) °C	*T*_g_ (II) °C
SEBS	11.0 ± 0.2	166.3 ± 1.3	4.0 ± 0.1	10.0 ± 0.1	99	101
SEBS-5G	21.1 ± 0.2	171.2 ± 1.4	5.8 ± 0.1	14.3 ± 0.3	102	99
SEBS-10G	33.1 ± 0.3	182.9 ± 1.4	9.3 ± 0.0	21.8 ± 0.2	101	100
SEBS-15G	40.7 ± 0.9	197.8 ± 0.6	12.4 ± 0.1	21.2 ± 0.2	100	100
SEBS-5GVS	20.4 ± 0.1	164.1 ± 0.9	5.6 ± 0.2	10.2 ± 0.1	97	97
SEBS-10GVS	36.5 ± 0.3	164.8 ± 0.6	10.2 ± 0.2	15.8 ± 0.1	99	101
SEBS-15GVS	54.5 ± 1.1	160.9 ± 1.1	15.4 ± 0.2	30.7 ± 0.6	103	97
